# Evaluating diabetes-related nutrition knowledge and dietary beliefs among type 2 diabetes patients in Vidarbha region, Maharashtra: a mixed method approach for insightful analysis

**DOI:** 10.3389/fnut.2025.1420662

**Published:** 2025-02-24

**Authors:** Umesh Kawalkar, Amar Mankar, Mahesh Puri, Anshu Singh, Shital Telrandhe, Abhay Gaidhane, Manoj Talapalliwar, Sayali Fuladi

**Affiliations:** ^1^Datta Meghe Institute of Higher Education and Research, Wardha, India; ^2^Government Medical College and Hospital, Akola, India

**Keywords:** diabetes mellitus, diabetes nutrition, diabetes nutrition knowledge, Vidarbha region, dietary beliefs

## Abstract

**Introduction:**

The purpose of this study is to evaluate diabetes-related nutrition knowledge and dietary beliefs among Type 2 Diabetes Mellitus (T2DM) patients in the Vidarbha region, Maharashtra, utilizing a mixed method approach. The study aims to assess the existing gaps in nutritional education and dietary practices among T2DM patients, with a specific focus on understanding gender-based differences in knowledge levels.

**Methods:**

A convergent parallel mixed method approach was employed at tertiary care hospital in Vidarbha region, over 18 months. Participants aged 30 years and above, diagnosed with T2DM for at least 6 months, were included. Quantitative data collection utilized structured questionnaires during interviews to assess socio-demographic characteristics and diabetes-related nutrition knowledge. Qualitative data collection involved in-depth interviews to explore participants’ perspectives on dietary beliefs and practices, ensuring ethical approval and informed consent.

**Results:**

Total 384 T2DM patients participated, revealing significant gender disparities in diabetes-related nutrition knowledge. Male participants exhibited higher awareness levels across various aspects of dietary recommendations. Quantitative analysis highlighted gender differences in knowledge regarding recommended fruit and vegetable intake, milk product consumption, types of fruits suitable for diabetics, inclusion of rice in diabetic diets, alcohol consumption, and recommended salt intake. Qualitative analysis identified key themes related to food choices, meal timing, portion control, dietary restrictions, and cultural influences.

**Conclusion:**

The study emphasizes gender-sensitive educational interventions to enhance diabetes-related nutrition knowledge and self-management practices among T2DM patients. Tailored interventions addressing gender-based knowledge gaps are crucial for improving diabetes management outcomes and overall health among T2DM patients in Vidarbha, Maharashtra.

## Introduction

Diabetes is a chronic, metabolic disease characterized by elevated levels of blood glucose (or blood sugar), which leads over time to serious damage to the heart, blood vessels, eyes, kidneys and nerves. The most common is type 2 diabetes, usually in adults, which occurs when the body becomes resistant to insulin or does not make enough insulin (1). Globally, the number of people with diabetes has increased from 382 million in 2013 to 592 million in 2023 and number is projected to more than doubled to 1.3 billion people by 2050, with most of those affected being in developing nations (2). With type 2 diabetes accounting for over 90% of cases, this sharp increase is closely linked to changing dietary and lifestyle choices characterized by higher energy intake and decreased physical activity—a trend that is parallel to the rising obesity epidemic of recent decades (3). India, widely known as the global centre for diabetes, reflects this alarming pattern, which is marked by a high percentage of undiagnosed cases that are frequently discovered only after complications arise (4, 5). In Maharashtra state prevalence of diabetes is 14.6% in urban and 10.7% in rural area among women, while 15.3% in urban area and 12.4% in rural area among men (6).

Despite advances in medical interventions, particularly the development of new antidiabetic drugs, combating diabetes remains difficult (7). The main source of the problem is the poor nutritional education and largely disregarded eating patterns of people with diabetes (8). It is essential to acknowledge the critical role that nutritional education plays, particularly in patients with Type 2 diabetes mellitus (T2DM) (9). This entails empowering people with diabetes-related nutrition knowledge encouraging healthy eating habits, such as sensible portion control and respect for suggested alcohol intake limits (10). In order to guarantee that nutrition education is effectively provided to people with type 2 diabetes, it becomes necessary to conduct a thorough evaluation of the gaps in the general public’s knowledge regarding diabetes-related nutrition (11).

Existing literature reveals a weakly positive relationship between nutrition knowledge and dietary intake, with a consistent association between higher knowledge levels and increased consumption of fruits and vegetables (12). However, the extent of this relationship in the context of T2DM remains relatively unexplored. Against this backdrop, the present study seeks to address this gap by examining diabetes-related nutrition knowledge using a validated instrument. Our focus is on a sample of T2DM adults managed in usual care settings, aiming to contribute valuable insights into the intricate interplay between nutrition knowledge and dietary behaviors within this specific population. In the Vidarbha region of India, where diabetes prevalence is significant, understanding the dietary beliefs and practices among type 2 diabetes mellitus (T2DM) patients is crucial for effective management. We quantitatively estimate diabetes related nutrition knowledge of T2DM adults managed in usual care using validated instrument with respect to gender. Our study also wants to gain deeper insights into the factors influencing dietary behaviors and identify potential areas for intervention and education. This study not only contributes to the existing body of knowledge on T2DM management but also provides context-specific insights that can inform tailored interventions to improve dietary practices and ultimately enhance the quality of life for T2DM patients in the Vidarbha region. So, with this background this study plans to evaluate the diabetes related nutrition knowledge and the dietary beliefs among the population of Vidarbha region with Type2 diabetes.

## Materials and methods

Convergent parallel mixed method approach study design was adopted to investigate diabetes-related nutrition knowledge among Type 2 Diabetes Mellitus (T2DM) individuals at a tertiary care hospital from Vidarbha region, Maharashtra, India. This hospital, provides treatment to patients referred from the four districts of Vidarbha region which includes Buldhana, Washim, Akola and Amarvati district. The study spanned 18 months and focused on individuals aged 30 years and above, diagnosed with T2DM for at least 6 months, and willing to participate. Exclusions comprised pregnant or lactating women, with other co-morbidities and those with intensive care unit admissions. Ethical Approval was taken from Institutional Ethics Committee of Government Medical College, Akola, and informed consent was obtained from each participant before data collection. The study adhered to the guidelines outlined in the Declaration of Helsinki, with approval from the Ethics and Medical Research Committee.

### Quantitative component

A sample size of 384 was calculated using a 95% confidence interval and a 10% allowable error, assuming a 50% prevalence of diabetes-related nutrition knowledge (13). Data collection involved a structured questionnaire administered through interviews, conducted in the local Marathi language. The questionnaire comprised socio-demographic like socioeconomic status was categorized on the basis of having Below Poverty Line (BPL) card issued by the competent government authority and personal characteristics, including age, sex, duration of diabetes, family history etc. Next section focused on diabetes-related nutrition knowledge, utilizing a pretested and predesigned questionnaire derived from a general nutrition knowledge questionnaire as per the National Institute of Nutrition (ICMR-NIN) dietary guidelines for Indian (14). The questionnaire was peer-reviewed by the experts such as public health experts, nutritional counselor, diabetic specialist and physician and piloted beforehand. It encompassed topics such as dietary recommendations, food groups, healthy food choices, and diet-disease and weight management. Random blood sugar (RBS) was also measured at time of data collection with glucometer manufactured by Morepen Laboratories Limited, with its validity ensured through regular quality control checks, calibration against certified reference materials, for accuracy and precision. Blood drop was drawn from the finger cleaned with alcohol swab and priced with the help of sterile lancet for RBS measurement.

### Qualitative component

The qualitative approach for this study involved conducting in-depth interviews with 22 diabetic patients with their consent to explore their perspectives on various themes related to dietary management. Participants were selected purposefully to ensure diversity in age, gender, and cultural backgrounds. Each interview focused on exploring the participants’ food choices, meal timing habits, portion control strategies, adherence to dietary restrictions, and the influence of cultural factors on their dietary practices. The data saturation meets after the 22 interviews as no new information or themes were emerged. Three trained interviewers which were post graduate students from Masters in Public Health (Nutrition) students were involved in data collection. All interviewers underwent a standardization process before conducting the interviews, which included familiarization with the questionnaire and regular monitoring to ensure consistency in how questions were asked. UK supervise and observed for the data accuracy and quality. Interviews were semi-structured and about 20–30 min. Participants were allowed to have the freedom to express their experiences and perspectives. Selected Participant group, ultimately help to provide rich insights into the lived experiences of diabetic patients in managing their diet.

## Data analysis

### Quantitative component

Analysis of data incorporated a standardized proforma for consistency and inter-observer agreement. Collected data was cross-checked and supervised. MS- excel is used for data entry, Epi info and Open Epi software were employed for analysis, along with duplicate entry to ensure data consistency. Simple proportions were calculated for each outcome indicator, and Chi-square test were applied to compare qualitative variables.

### Qualitative component

In data analysis was done manually, AM oversaw the thematic analysis process, ensuring consistency and accuracy across all stages: familiarization, coding, theme identification, theme review, theme definition, and reporting. SF conducted initial data coding, identifying key themes related to food choices, meal timing, portion control, dietary restrictions, and cultural influences. UK provided expertise in qualitative research methods, contributing to the refinement of themes, their review and definition, and the interpretation of findings, ensuring a comprehensive and robust analysis.

## Results

The study was conducted at the tertiary care hospital, aiming to explore the diabetes-related nutrition knowledge of T2DM adults managed in usual care. A total of 384 patients actively participated in the study.

The socio-demographic characteristics of the study participants revealed interesting trends from Table 1. The majority belonged to the age group of 51–60 years, constituting 159 (41.47%). The gender distribution favored males, comprising 234 (60.9%) of the participants. Urban residents constituted a substantial portion, accounting for 281 (73.2%) of the sample, while the majority were married 262 (95.1%). Hinduism was the predominant religion among the participants, with 262 (68.3%), and a significant proportion belonged to lower socio-economic classes, i.e., Below Poverty Line (BPL) 234 (60.9%). Examining the characteristics related to diabetes, it was observed that a considerable number of participants had been living with diabetes for 3–6 years 122 (31.7%). Fasting blood sugar levels were predominantly uncontrolled (>130 mg/dL) in 240 (62.6%) of cases. The majority managed their diabetes through oral medication 253 (65.9%), with a smaller percentage on insulin therapy or a combination of oral and insulin treatment.

**Table 1:** Baseline characteristics of study participants.

Socio-demographic factors	*n*: 384 (%)
Age (years)	
31–40	28 (7.31%)
41–50	47 (12.2%)
51–60	159 (41.47%)
>60	150 (39.02%)
Sex	
Male	234 (60.9%)
Female	150 (39.1%)
Residence	
Urban	281 (73.2%)
Rural	103 (26.8%)
Marital status	
Married	365 (95.1%)
Single (Widow/Divorced/Single)	19 (4.9%)
Religion	
Hindu	262 (68.3%)
Muslim	93 (24.4%)
Other	29 (7.3%)
Socio-economic status	
Above poverty line (APL)	150 (39.1%)
Below poverty line (BPL)	234 (60.9%)
Duration of diabetes (years)	
<3	94 (24.4%)
3–6	122 (31.7%)
6–10	66 (17.1%)
>10	102 (26.8%)
Fasting blood sugar (mg/dl)	
Controlled (≤130)	88 (22.8%)
Uncontrolled (>130)	240 (62.6%)
Not knowing	56 (14.6%)
Management	
Oral medication only	253 (65.9%)
Insulin only	19 (4.9%)
Combination (oral+ Insulin)	112 (29.2%)

The evaluation of knowledge related to diabetes and nutrition revealed notable differences among participants, particularly in terms of gender as mentioned in Table 2. Male participants demonstrated significantly higher awareness levels compared to their female counterparts in various aspects. For example, males showed better understanding of recommended fruit and vegetable intake (40% compared to 25% in females, *p* = 0.03), as well as milk product consumption (40% compared to 13% in females, *p* = 0.02). Similarly, males exhibited greater knowledge regarding the types of fruits suitable for diabetic individuals (24% compared to 13% in females, *p* = 0.0001) and the inclusion of rice in diabetic diets (64% compared to 50% in females, *p* = 0.0001). However, both genders showed similar levels of understanding regarding alcohol consumption (88% overall) and the recommended salt intake for diabetic patients (75% overall).

**Table 2:** Diabetes related nutrition knowledge among study participant.

Diabetes related nutrition knowledge	Correct answer (*n*: 384) (%)	Male (*n*: 234) (%)	Female (*n*:150) (%)	*p* value*
1. How many times in one week a diabetic patient should eat fruits and vegetables?	131 (34%)	94 (40%)	37 (25%)	0.03
2. How many times in a week diabetic patient should consume milk and milk products?	113 (29%)	94 (40%)	19 (13%)	0.02
3. Which type of fruits the diabetic patient should eat?	75 (20%)	56 (24%)	19 (13%)	0.0001
4. Whether diabetic patients should include rice in their meals?	225 (58.5%)	150 (64%)	75 (50%)	0.0001
5. Should a diabetic patient drink alcohol?	338 (88%)	225 (96%)	113 (75%)	0.0001
6. In how much quantity salt should be eaten by patients suffering from diabetes?	290 (75%)	215 (92%)	75 (50%)	0.0001

*Chi-square test.

The thematic analysis (Table 3) of qualitative interviews conducted among study participants revealed several key findings regarding their dietary beliefs. Participants emphasized a strong inclination toward traditional food preferences, such as jowar (*Sorghum*), bajri (*pearl millet*), and tur (*red gram*), alongside a preference for locally sourced ingredients, attributing them with freshness and superior taste. As one participant remarked, *“I prefer locally grown vegetables and grains because they are fresh and healthier.”* Additionally, participants expressed a consensus regarding the importance of early dinner consumption for controlling blood sugar levels, reflecting a belief in the direct impact of meal timing on glycemic control. A participant highlighted this, saying, *“I eat dinner early, around 7 pm, to manage our sugar levels better.”* Portion control emerged as a prominent strategy, with participants employing visual cues and individualized approaches to manage portion sizes effectively. One participant stated, *“I use smaller plates to control portions and prevent overeating.”* However, adherence to dietary restrictions posed significant challenges, particularly concerning sugar and carbohydrate intake, exacerbated during social gatherings where maintaining dietary discipline proved arduous. *“It’s hard to resist sweets and snacks during festivals and parties,”* lamented a participant, reflecting on the challenges they faced. Cultural influences were cited as pervasive, shaping food choices and consumption patterns profoundly. “*I adhere to the practice of having dates during Ramadan as they provide a quick source of energy, but cautious about the portion sizes due to diabetes,”* emphasized a Muslim participant, highlighting the intersection of cultural traditions with dietary considerations among diabetic patients in the Vidarbha region. Another participant remarked, “Our culture emphasizes rich, flavorful food, which can be difficult to avoid, even with diabetes.” Furthermore, participants predominantly relied on healthcare providers, particularly doctors and dietitians, for guidance on dietary management, highlighting the pivotal role of medical professionals in shaping dietary beliefs and practices among T2DM patients.

**Table 3:** Qualitative findings on dietary beliefs among study participants.

Theme	Sub-theme	Description	Representative quote	Interpretation
Food choices	Traditional food preferences	Most of the study participants said that they prefer traditional foods such as roti, dal, and vegetables, reflecting cultural dietary habits.	*I eat roti, dal and vegetable as it feels like complete meal.*	Cultural and familial influenced majorly contribute to diet preferences and practices in daily food consumptions.
	Preference for locally sourced ingredients	Locally sourced ingredients like fresh vegetables and fruits were favored due to their perceived health benefits and availability in the region by study participants	*I eat locally grown vegetables and grains like spinach, brinjal, jowar, wheat because they are fresh and healthier.*	It highlights the trust in local faming practices and belief of high-quality produces.
Meal timing	Early dinner preference	Many individuals exhibited a preference for early dinners, believing it helps in better blood sugar control and digestion.	*I eat dinner early, around 7 pm, to manage sugar levels better*	This reflects the belief of meal timing is deeply rooted in the society as the measure to control the glycemic levels.
	Belief in controlling blood sugar levels	There was a prevalent belief among many study participants that timing meals according to blood sugar levels can help manage diabetes effectively, influencing meal timing choices.	*I consume some food every two-three hourly. It help to keep my sugar levels under control*	Awareness of meal timing in glycemic control indicates knowledge about medical perspectives among population.
Portion control	Individualized portion management	Portion sizes were often adjusted based on individual preferences and perceived hunger levels, indicating a personalized approach to portion control.	*I use smaller plates to control portions. I am avoiding to eat some heavy foods like samosa; kachori; ghee etc.*	The individuals prefer different methods or approaches for the portion control in order to maintain ideal diet and glycemic levels.
Dietary restrictions	Struggle with sugar and carb intake	Majority of study participants talked about facing challenges in limiting sugar and carbohydrate intake, especially with traditional sweets and carb-rich foods deeply ingrained in local festivals.	*It’s hard to resist sweets and snacks during festivals and parties.*	Emotional or behavioral psychological factors influenced the dietary restrictions among the individuals
	Challenges during social gatherings	Social gatherings present challenges as patients may encounter pressure to consume foods not aligned with their dietary restrictions, leading to dilemma told by study participants.	*In family functions like marriage; birthday parties or festivals like Diwali; Hoi it is difficult to say no to sweets and fried foods*	Social and peer pressure leading to poor adherences to the dietary practices especially at gatherings.
Cultural influences	Influence of cultural practices on diet	Cultural practices heavily influence dietary choices, with traditional beliefs shaping food preferences and meal habits among diabetic patients.	*I adhere to the practice of having dates during Ramadan as they provide a quick source of energy, but I am cautious about the portion sizes due to diabetes*	There strong cultural significance in dietary practices among the people with different cultural follows different diets and practices irrespective of its glycemic index
	Integration of modern dietary advice	While traditional practices dominate, there’s an increasing integration of modern dietary advice, reflecting a shift toward incorporating medical recommendations.	*I follow my doctor’s advice but still eats traditional sweets like puran-poli during festive seasons*	The integration of modern dietary advice alongside traditional practices highlights a gradual adaptation of medical recommendations by individuals but with selective adherence influenced by cultural preferences

## Discussion

The results of the research we conducted reveal notable variations, in diabetes-related nutrition knowledge between male and female participants, highlighting the need for gender-specific educational interventions in managing Type 2 Diabetes Mellitus (T2DM). Our results are consistent with previous research, supporting earlier studies conducted in different regions that also identified gender-based disparities in diabetes knowledge and self-management practices. Our study did not prescribe or evaluate a single definition of an optimal diabetes diet. Instead, we adhered to the ICMR-NIN dietary guidelines for Indians, which provide evidence-based recommendations tailored to the Indian context. These guidelines emphasize culturally appropriate, region-specific, and balanced dietary choices for individuals with diabetes.

Our study revealed significant gender differences in DRNK, with male participants demonstrating higher knowledge across multiple domains, including recommended fruit and vegetable intake, milk product consumption, and types of fruits suitable for diabetics. This aligns with findings from Han et al. (11) who emphasized the importance of culturally tailored and gender-specific nutrition education programs to address these gaps. Similarly, Mankar et al. (15) highlighted that gender-specific factors, such as access to education and healthcare, significantly influence glycemic control in Indian populations. Similar to prior research in Bangladesh, our study reveals a widespread lack of knowledge among T2DM patients concerning the causes, management, and risk factors of diabetes, despite receiving professional health education (16). Likewise, studies in Thailand and Saudi Arabia have shown poor adherence to dietary recommendations among T2DM patients, indicating difficulties in translating knowledge into action (17). These results highlight the critical need for better educational initiatives catered to the unique requirements of individuals with type 2 diabetes, particularly in addressing gender-based differences. Cultural preferences and beliefs emerged as prominent themes influencing dietary practices among T2DM patients. Traditional foods, such as jowar and bajri, were favored, reflecting a reliance on locally sourced ingredients. This finding echoes the work of Leblanc et al. (18), who noted that cultural dietary patterns significantly shape adherence to dietary interventions. However, challenges, including adherence during social gatherings and cultural events, were evident. Blanks et al. observed similar challenges in their study of African American women, where social and cultural contexts shaped dietary behaviors and intervention outcomes.

In contrast, studies in China and Singapore have demonstrated the effectiveness of targeted educational interventions in improving diabetes-related nutrition knowledge and self-management behaviors among T2DM patients (19). Educational programs in China led to significant enhancements in nutrition knowledge and practice accuracy, resulting in better glycaemic control (19). Similarly, although no direct link was found between knowledge and diet quality in Singapore, barriers and facilitators to dietary guideline adherence were identified, emphasizing the importance of comprehensive educational interventions (11). Nutrition education plays a pivotal role in bridging knowledge gaps and improving self-management among T2DM patients. The systematic review by Karkhah et al. (20) found that higher education levels and family history of diabetes were positively correlated with better DRNK. Our findings, where urban male participants demonstrated higher knowledge, further emphasize the need for education tailored to rural and female populations. Furthermore, Ramadas et al. (21) and Ahmed et al. emphasized the role of structured educational interventions in bridging these gaps, demonstrating improvements in dietary practices and glycemic control post-intervention.

Additionally, studies conducted in Trinidad and Tobago demonstrated the critical impact that dietary guidance has in improving diabetes knowledge, attitudes, and practices among patients (22). Patients who received counseling demonstrated better knowledge and adherence to dietary recommendations compared to those who did not receive counseling. Barriers, such as lack of time, stress, and conflicts between personal values and medical advice, were consistent with global studies. Han et al. (11) identified similar barriers in Singapore, emphasizing the need for personalized and context-specific interventions to improve dietary adherence. Furthermore, Auryan and Itamar (23), stressed the importance of addressing gender-specific challenges, particularly among women who face unique physiological and socio-economic barriers. This emphasises how important personalized education and counseling are in improving diabetes-related outcomes.

In comparison to these studies, our research makes a contribution by identifying the precise domains of diabetes-related nutrition knowledge where gender differences are present. Male participants showed higher awareness levels compared to females in various aspects of dietary recommendations for diabetes management but reasons for this is not studied in our research. It might be due to the cultural or other factors like education that need to be explored in further future research. These variations highlight the requirement for customized educational interventions that address gender-specific knowledge gaps and empower all T2DM patients to make informed dietary choices for better disease management.

The study conducted by Colles et al. (24) among a group of older, well-educated Asian Indians residing in urbanized regions of New Delhi shed light on various dietary patterns and beliefs prevalent among individuals with Type 2 diabetes mellitus (T2DM). Despite recommendations for higher consumption of fruits and vegetables, the average daily intake among respondents remained low, with a preference for processed meals and snacks being observed. Interestingly, those who reported eating according to what they were served displayed lower consumption of fruits and vegetables, indicating a potential lack of personal food choice responsibility. Additionally, erroneous beliefs regarding the health value of certain foods, particularly processed snack foods, were associated with higher consumption frequency. The study emphasized the importance of personalized dietary education and active participation in diabetes self-management, highlighting the need for interventions targeting cultural influences and promoting healthier food choices. These findings echo those of other studies, such as the one conducted in Southern India, which also reported poor knowledge about diabetes management, especially among females and those less educated (25). Furthermore, the study revealed the common use of high-fat ingredients like butter and desi ghee, suggesting a need for public health interventions aimed at reducing saturated fat intake and increasing awareness about healthier dietary practices. Despite its limitations, including the reliance on memory for dietary reporting and the small sample size, this study provides valuable insights into the dietary beliefs and behaviors of T2DM patients in urban North India, underscoring the necessity for tailored dietary interventions to improve metabolic health outcomes in this population. The exclusion of LGBTQ+ individuals, whose unique dietary beliefs and access to healthcare may differ from the general population. Additionally, individuals with other comorbid conditions were not included, which may limit the generalizability of the findings to those with complex health profiles that require specialized dietary and medical interventions.

In conclusion, our study highlights the importance of gender-sensitive educational interventions in improving diabetes-related nutrition knowledge and self-management practices among T2DM patients. By addressing the knowledge gaps between genders and offering specialized education and counseling, healthcare providers can effectively enhance diabetes management outcomes and promote better overall health and well-being among T2DM patients.

## Data Availability

The raw data supporting the conclusions of this article will be made available by the authors, without undue reservation.
